# Artificial Intelligence and Corruption: Opportunities and Challenges in the Health Sector

**DOI:** 10.1002/hpm.70002

**Published:** 2025-06-11

**Authors:** Paula del Rey‐Puech, Dina Balabanova, Martin McKee

**Affiliations:** ^1^ Faculty of Public Health and Policy London School of Hygiene & Tropical Medicine London UK

**Keywords:** artificial intelligence, corruption, healthcare, machine learning

## Abstract

Corruption in health systems diverts resources, erodes trust, and reduces service quality. Traditional oversight methods struggle to detect fraudulent patterns, but Artificial Intelligence (AI) offers new possibilities. AI can analyse large datasets to predict corruption risks and detect irregularities in procurement, insurance claims, and counterfeit medicines. Successful applications include AI‐powered tools that flag suspicious transactions, expose bid‐rigging in procurement, and identify fraudulent medical billing. AI can also complement other analytical tools to help track counterfeit drug supply chains through image recognition and network analysis. However, AI's impact depends on how it is deployed. Government‐led AI initiatives may enhance transparency but risk reinforcing power imbalances or enabling authoritarian control. In contrast, civil society‐driven efforts can empower citizens to hold authorities accountable but face challenges like limited data access and misinformation risks. Moreover, AI can also facilitate corruption in the health system through biased algorithms, deepfake propaganda, or manipulated AI‐driven decision‐making in resource allocation. Maximising AI's anti‐corruption potential in healthcare requires investments in skilled personnel and data systems. AI should complement human oversight, with transparent auditing mechanisms to mitigate biases. Integrating blockchain and AI technologies may enhance accountability by securing procurement records and preventing data manipulation. While AI presents significant opportunities, its application to anti‐corruption remains a political issue as much as a technological one. Careful governance, ethical and legal safeguards, and balanced implementation will determine whether AI combats corruption or exacerbates abuses.

**Summary**

- AI offers innovative ways to detect hidden corruption in health systems
- Examples of its use include procurement, insurance billing, and medicine supply
- It requires investment in data, skills, transparency, and accountability
- The political, ethical and legal contexts influence whether it is a force for good or bad

## 1 Introduction

Corruption poses a serious threat to health and health systems worldwide [1]. It diverts scarce resources away from essential care, undermines trust in institutions, and compromises the quality of medical services. Yet, it is often virtually invisible as corrupt behaviour occurs in the shadows, with its perpetrators working to conceal their actions, leveraging bureaucratic complexity and opaque financial systems to evade scrutiny. The evidence trail is often hidden inside large datasets, such as procurement records or insurance claims, making fraudulent patterns difficult to identify using traditional oversight mechanisms.

Artificial Intelligence (AI), particularly recent advances in deep learning and generative AI, hold significant potential to support anti‐corruption efforts in the health sector. AI systems, such as multimodal deep learning models, can process large volumes of structured and unstructured data across diverse modalities (e.g., text, images) at speeds far exceeding human capacity. Unsupervised machine learning methods, which rely on unlabelled data, can reduce training costs and help uncover previously unknown patterns. Unlike traditional machine learning, deep learning models do not require manual specification of features of interest; they learn hierarchical representations directly from raw data [2]. These enable the identification of complex, non‐linear patterns useful for tasks such as anomaly detection, classification, and predictive modelling [3]. Applications include: detecting irregularities in procurement transactions, estimating the likelihood of fraudulent activity, or identifying the most salient risk factors associated with corruption. Additionally, generative AI models, such as large language models (LLMs), can efficiently examine and extract insights from large textual datasets [4], which may reduce reliance on manual document checks, a process susceptible to corruption practices.

These capabilities have informed the development of AI anti‐corruption tools (AI‐ACT) to combat corruption in other sectors (Table 1) [5]. Odilla has described 31 initiatives in Brazil that have used AI‐powered bots to mine public datasets and flag suspicious transactions covering budget allocations, expense reports, and procurement contracts [6]. For example, ‘Rosie’, an AI bot developed by civic hackers, scanned Brazilian congressional expense claims and alerted the public to potential fraud, for example, a member of Congress claiming reimbursement for a meal consisting of 12 kg of food. However, while several were used to identify irregularities in procurement in general, only a small number focused on the health sector, primarily examining COVID‐19‐related contracts. AI models have also been used in Italy to predict which municipalities would likely experience corruption crimes, which can allow authorities to prioritise anti‐corruption efforts [7]. Additionally, analysis of economic and political data from Spanish provinces identified risk factors for corruption, such as rapid economic growth, real estate booms, and a single party in power for extended periods [8]. Machine learning tools can search social media posts to find accounts of experiencing corruption [9]. The ability of AI to learn rapidly is critical, as those engaged in activities such as money laundering must constantly adapt their methods to avoid detection, something that traditional rule‐based systems may struggle with [10].

**TABLE 1:** Illustration of potential applications and functionalities of AI‐based anticorruption tools (ACT‐AI) and other complementary analytical and technological tools in the healthcare sector.

Area	Anti‐corruption tool	Application	Functionality (examples)
Procurement monitoring	Machine learning	Pre‐bidding, bidding, post‐bidding phases	Detect irregular pricing and bid rigging, predict implementation inefficiencies
	Network analysis	Vendor relationships	Identify collusion and cartel behaviour
	Blockchain integration	Procurement records	Ensure transparency and tamper‐proof documentation
	Natural language processing	Legal and procurement documents	Automate review and flag suspicious clauses or contract modifications
	Citizen reporting platforms	Citizen engagement	Enable the public to flag irregularities and monitor contracts
Health insurance & billing oversight	Machine learning	Health insurance claims data	Detect fraudulent patterns, flag novel fraud patters
	Multimodal data integration	Cross‐referencing claims with external data	Enhance fraud detection using social media, geolocation, and usage patterns
Medicine supply chain	Deep learning	Counterfeit drug detection	Compare packaging to reference databases
	X‐ray fluorescence and machine learning	Drug composition analysis	Identify substandard or fake medicines
	Network analysis	Pharmaceutical supply chains	Map and detects counterfeit drug networks
Governance & oversight	Predictive analytics and machine learning	Risk profiling	Identify high‐risk contracts, municipalities, or officials
	AI bots for data mining	Budget and expense monitoring	Flag and report suspicious transactions and spending
	Satellite imagery	Infrastructure and remote monitoring	Detect ghost projects and environmental crimes
Ethical & legal safeguards	Algorithm auditing and bias monitoring	All AI systems	Ensure fairness, transparency, and accountability
	Human‐in‐the‐loop systems	Decision support	Combine AI insights with human judgement for final decisions
	Responsible AI frameworks	Policy and governance	Guides ethical development, deployment and oversight of AI tools

## 2 Tackling Corruption in Health Systems

Health systems are susceptible to many types of corruption [11]. Here, we consider three areas where corruption is thought to be particularly widespread and which illustrate what is possible with AI and complementary analytical approaches: procurement, insurance billing, and counterfeit medicines, as well as two overriding themes, governance/oversight and ethical/legal safeguards (Table 1). We stress that these are illustrative, and there is potential for applying AI in other areas, although such applications remain less well developed. We also argue that if the potential of AI‐ACT is to be realised, certain prerequisites should be put in place, with political, legal and ethical considerations needed to guide how these tools are used and not misused [5].

### 2.1 Procurement

Procurement offers particularly lucrative opportunities for corrupt actors, including bid rigging, insider deals, and fraudulent contract execution. Traditional oversight methods often fail due to human ingenuity in circumventing them. AI offers opportunities to improve procedures across procurement phases: pre‐bidding, bidding, and post‐bidding, but also to engage citizens in the accountability process [12, 13]. Del Sarto et al. describe profiling healthcare contracting authorities to find indicators of risk of corruption [14].

Corruption in the pre‐bidding phase includes manipulated tenders, insider leaks, and biased vendor selection. Machine learning techniques can help detect irregular pricing strategies and assess vendor reliability using historical performance data to identify deviations from norms [15]. Network analysis can detect cartels in clusters of firms with unusually dense connections that align with bid‐rigging rings [16].

Bribery and bid manipulation occur in the bidding phase. Machine learning algorithms can detect irregularities in patterns suggestive of collusion or fraudulent behaviour [15]. Blockchain technology can facilitate the transparency and security of transactions [17], thereby enhancing the integrity and reliability of data used by algorithms.

Corruption in the post‐bidding phase includes inflated invoices, contract modifications, and subpar project delivery. Machine learning models have helped predict which public procurement contracts lead to investigations, contract breaches or weak implementation [18]. Natural language models can reduce human involvement in contract review and management [19]. The AI‐powered national system, PROZORRO, enables citizens, civil society, and journalists in Ukraine to monitor and analyse public contracts [13]. They can report irregularities and raise red flags when they find suspicious patterns, such as vendor favouritism or inflated pricing.

### 2.2 Insurance/Billing Issues

A recent systematic review describes how AI offers the potential to detect anomalies in health insurance claims [20]. Supervised machine learning models can be trained on labelled datasets of fraudulent or non‐fraudulent claims [21] but, as much fraud may be undetected, and therefore unlabelled, unsupervised models may be better at identifying outliers and flagging unusual patterns [22]. Moreover, advanced AI systems can integrate diverse data, such as news articles, social media posts, geolocation information, IP addresses, and usage patterns, to uncover connections that might otherwise remain undetected [23].

### 2.3 Corruption in Medicines Supply

Medicines are implicated in several forms of corruption: diversion of supplies to private markets, procurement (as discussed above), and supply of counterfeit or substandard products [24]. These are areas experiencing rapid technological advances, creating new opportunities [25].

AI tools can identify counterfeit medicines by comparing packaging to reference databases. Ferdosi et al. reported up to 96% accuracy in brand recognition and 84% in fake detection using a pre‐trained convolutional neural network model [26]. Other approaches involve combining machine learning algorithms with x‐ray fluorescence to analyse the chemical composition of drugs [27]. AI‐SNIPS, involving joint AI and network analysis, has been proposed to identify counterfeit drug networks [28], but such systems remain vulnerable to attacks from subtle, intentional manipulations of input data, highlighting the importance of ongoing vigilance [29].

### 2.4 The Power Dynamics of AI‐ACT

These examples illustrate the potential for using AI‐ACT in the health sector. However, technological advances often change the distribution of power in society. Thus, Köbis and colleagues [5] distinguish between top‐down and bottom‐up approaches. Proponents of the former argue that AI is impartial, and having ‘no human in the loop’ reduces biases and influence of vested interests. However, critics warn that top‐down AI applications risk entrenching existing power structures rather than dismantling corruption. In authoritarian states or those with a weak rule of law, AI can become a tool for political control, selectively targeting opposition figures while shielding government‐aligned individuals. AI‐powered governance can be simultaneously used to improve public services and for state surveillance, for examply by using AI facial recognition [30]. Even in settings like the US, where AI‐driven facial recognition is legally limited, other AI tools are being developed to recognise people using other attributes such as body size [31].

Hence, the opportunities and risks associated with the ability of single institutions to access large‐scale individual data, combined with powerful AI tools, should be carefully balanced. For example, China's Zero Trust programme, which accessed over 150 databases to monitor public officials' transactions, proved effective in detecting suspicious activity. However, it also triggered backlash over privacy and surveillance concerns, and the system was ultimately discontinued [32]. Similar concerns about data access and trust could arise in health systems with expanding AI‐driven oversight.

In contrast, bottom‐up approaches leverage AI to empower civil society by helping to scrutinise government actions [6, 33]. These approaches can shift power dynamics, especially where state institutions are untrustworthy or compromised. Nevertheless, as noted by Odilla [6], access to data is often restricted. Citizen‐led AI initiatives require sustained engagement, but many digital activism movements struggle with longevity and scalability. Furthermore, AI‐driven public exposure of corruption carries risks of misinformation, wrongful accusations, and reputational harm when AI systems make classification errors. There is also the danger of populist movements, including those supported by foreign states and subnational economic and political networks, misusing AI tools to target individuals unfairly.

For these reasons, the role of AI‐driven anti‐corruption efforts depends on who controls the data and algorithms. When deployed by governments, AI can either reinforce accountability or entrench authoritarianism. When used by citizens, AI can serve as a tool for democratic oversight, but it faces many challenges.

In health systems, power is often concentrated in hierarchical structures where decision‐making authority is unequally distributed. This creates fertile ground for corruption to persist undetected, particularly in settings with a weak rule of law. Cultural and institutional barriers compound these structural vulnerabilities. Fear of retaliation, weak legal protections, and cultural norms already constrain the effectiveness of whistleblowing mechanisms in health organisations [34]. In such contexts, deploying AI to reduce corruption may unintentionally reinforce harmful power dynamics, especially if implemented without appropriate legal frameworks, independent oversight or stakeholder consultation.

Using AI in the healthcare sector also presents an important ethical tension. Clinical decision‐making still largely has a ‘human in the loop’ model, where health professionals are expected to exercise discretion, empathy, and professional judgement. Other concerns include how, in public health systems, AI monitoring procurement contracts, clinical performance, or resource allocation, without strong governance arrangements, may selectively penalise frontline workers while shielding high‐level mismanagement. In private healthcare, AI systems that detect fraudulent billing or overprescribing may prioritise protecting corporate profitability rather than patient welfare.

## 3 AI as an Enabler of Corruption

As noted earlier, the use of AI‐ACT involves institutional and political risks. Consequently, public bodies should be aware of and, as far as possible, take measures to reduce them [35].

AI systems can be programed to serve corrupt interests. For instance, political leaders or businesses may commission AI‐generated deepfakes to discredit opponents or manipulate public opinion. Similarly, AI‐driven propaganda bots can flood social media with disinformation, amplifying political or financial agendas (such as manipulating share prices) while concealing the identities of those behind the manipulation. Disinformation around vaccines and public health messaging in healthcare has been particularly salient since the COVID‐19 pandemic.

Other research has described the motivations of those carrying out adversarial attacks on AI systems [29], for example, to force machine learning models to make mistakes. In healthcare, this can have catastrophic consequences for patients, such as missing counterfeit drugs or wrongly denying treatment insurance claims.

What makes AI‐enabled corruption particularly dangerous is its scale and opacity. Unlike traditional corruption, which typically involves direct human interactions, AI can automate and obscure illicit activities, making them harder to trace. Furthermore, AI's complexity allows perpetrators to deny responsibility, blaming algorithmic ‘errors’ or data biases.

## 4 What Does This Mean for Health Policy?

The development and deployment of AI systems require careful consideration of the level of human involvement at each stage of the decision‐making process. If public bodies are to use it, they must invest in the people who will interact with it. This means training and retaining appropriately skilled analysts and investigators, whose scarcity is often a significant barrier to innovation [36]. Investment is also needed in data systems to train and apply the algorithms [37]. The challenges are especially significant in low‐income settings where providing competitive salaries will be extremely difficult. Even the best AI algorithms will be useless without effective governance and relevant parties must adhere to ethical and legal frameworks that protect the public from its risks [38].

AI systems rely on high‐quality, comprehensive data to function effectively. Odilla's research in Brazil described the lack of access to official data thwarting civil society's efforts, such as limiting the ability to extend use of these tools to other regions and certain public institutions [6]. Data fragmentation, missing records, complex regulation, and inconsistent reporting practices in many healthcare systems pose significant barriers to implementation. The limited number of fraudulent cases identified in many data sets also complicates training machine learning models using supervised methods [20]. Unsupervised methods, which do not require labelled data, can address some of the limitations of supervised approaches and offer the potential to identify previously unknown anomalies and patterns within datasets. Although progress in applying these methods has been gradual, there are promising examples in the healthcare sector. For instance, De Meulemeester et al. developed a workflow that applied unsupervised anomaly detection techniques to the behaviour of healthcare practitioners obtained from healthcare insurance data in Belgium. The approach outperformed traditional detection methods and successfully identified a previously unrecognised anomalous pattern among general practitioners [22].

Efforts to standardise healthcare data, enhance interoperability, and promote open data initiatives will be crucial to optimise the potential of AI to detect corruption. Breaking down data silos (e.g., linking company registries with contract data and asset declarations) can dramatically increase the ability to detect complex fraud webs and some scholars call for investments in ‘accountability data’, like asset disclosures and political finance records, to train AI models [37]. Legal frameworks should facilitate responsible data sharing since many citizen‐led tools rely on open data access [6].

The ability of corrupt actors to deliberately manipulate or withhold data poses an obvious challenge. AI systems can enhance the capabilities of existing technologies, such as blockchain, which can help by creating tamper‐proof vendor records, ensuring fair and transparent procurement decisions. This involves the creation of ‘blocks’ of time‐stamped transaction data from multiple trusted parties connected by a chain. A complete picture of the transaction is visible only when these blocks are brought together. Dispersal of the blocks across multiple servers prevents any one person from manipulating it [39]. Several blockchain‐based systems have been implemented to enhance drug supply chain security [40], and AI systems can further amplify their capabilities [41].

If AI is to maximise benefits and minimise harm, it must adhere to principles of transparency, fairness, and accountability [38]. False positives can have enormous consequences for officials, providers and patients. For example, nearly half of the AI‐ACTs used to monitor public expenditure were identified in Odilla's research as underused, partly due to a high incidence of false positives. Thus, if an AI application flags an official as high‐risk, there should be transparent procedures for review to enable the official to challenge the finding. In Brazil, while the technology behind Rosie proved effective, the evidence generated was not deemed sufficient to support prosecution in many cases, highlighting the complex regulatory framework that bottom‐up initiatives have to navigate [42]. Responsible use also entails monitoring AI performance for bias. Another AI‐driven tool, Mara, developed in Brazil, helps predict corruption within the civil service but faces criticism that it reinforces biases, given that it was trained on data on individuals who had been previously punished for corruption [42]. Periodic audits of the models can ensure they are not disproportionately flagging one demographic or systematically overlooking certain elites. Public authorities must also recognise and protect themselves from those who exploit AI to engage in corruption.

While minimising human involvement is one of the premises in advocating for AI‐ACT, it is vital to strike a balance in the AI‐human collaboration, especially in the healthcare sector. Legal, ethical, and operational frameworks should guide decisions about where human oversight is needed in the AI pipeline and to what extent. This is especially important for AI agents, which can autonomously interact with the environment and execute actions [43]. AI efficiencies in sifting data and pinpointing anomalies should be balanced with the context, ethical judgement, and legal expertise of humans to prosecute or sanction corruption. In many cases, AI can be used as a decision‐support tool [6]. For instance, an AI system might generate a risk score for each public contract; rather than acting automatically, it would hand over the top‐risk contracts to human auditors for deeper examination. This approach leverages the strength of both AI's speed and pattern recognition with human intuition and scepticism. It also helps build trust in AI, as users see it helping them rather than making unchecked decisions.

As AI tools prove their worth in pilot projects, scaling them up sustainably is the next step. For example, despite Rosie's success in identifying over 8000 suspicious transactions and an initially successful crowdfunding campaign, operational costs have limited the team's ability to sustain the tool solely with voluntary efforts [44]. Governments should institutionalise successful tools so they persist beyond a single political administration or donor project. Linking or integrating them into existing structures and processes, for example, through hybrid AI‐human models, is key. For example, one of the successes behind the AI‐driven bot Alice, which helps to analyse public procurement transactions and identify errors or fraud in Brazil, has been its integration into existing workflows at the federal office of the Comptroller General (CGU) [45].

There are also promising new frontiers for AI in anti‐corruption that merit exploration. One area is the use of satellite imagery and remote sensing—AI analysis of satellite data can bring transparency to remote locales, detecting activities like illegal mining, environmental crimes, or ghost infrastructure projects [37]. Another emerging area is utilising AI to manage large‐scale citizen feedback or complaints, identifying systemic issues from crowdsourced information, ensuring these feed seamlessly into system change [37]. For example, platforms such as Pol.is, which employ AI methods to collect and analyse public opinions, have been used to promote online deliberation and facilitate consensus‐building [46, 47]. Further research into how such platforms could be adapted to foster dialogue and raise awareness about corruption in the healthcare sector would be valuable, particularly in supporting participatory approaches to foster transparency and accountability.

However, mobilisation of the public against corruption is not easy, not least because of the risks to those who speak out. Starke and colleagues used conjoint analysis to test different messages for their ability, when incorporated in bots, to motivate users to call out corruption [48]. This revealed differences in what motivates people in different demographics, with some driven mainly by the severity of corruption, whereas this is less important for others. Importantly, using the word ‘corruption’ makes little difference. By exploring these frontiers, the anti‐corruption community can stay ahead of perpetrators who constantly seek new loopholes. However, AI‐ACT is a mechanism to detect and predict corruption, and it does not address the systemic and political factors that lead to corrupt practices in the healthcare sector in the first place. Systems must constantly adapt to prevent and tackle corruption without addressing these drivers.

## 5 Conclusion

AI holds immense promise for detecting corruption in the healthcare sector, drawing on proven methodologies from finance, public administration, and law enforcement. AI can uncover fraudulent activities that would otherwise go unnoticed by analysing procurement data, medical billing records, and financial transactions. However, realising AI's full potential requires addressing key challenges, including data limitations, algorithmic bias, and a lack of capacity to integrate it into existing systems.

Combined with strong institutional governance, human oversight, legal frameworks and ethical AI principles, AI can be a powerful force in promoting transparency, accountability, and integrity in healthcare systems, especially when used from the bottom‐up. However, with authoritarian regimes that are themselves engaged in corruption, it can also be a means to suppress criticism. For these reasons, increasing AI‐ACT is as much a political as a technological issue.

## Conflicts of Interest

M.M. and D.B's work on corruption and M.M's work on AI are supported by the European Observatory on Health Systems and Policies. P.d.R.‐P. is employed by the NHS in England.

## Data Availability

The authors have nothing to report.

## References

[1] E. Hutchinson , D. Balabanova , and M. McKee , “We Need to Talk About Corruption in Health Systems,” International Journal of Health Policy and Management 8, no. 4 (April 2019): 191–194, 10.15171/ijhpm.2018.123. 31050963 PMC6499907

[2] I. Goodfellow , Y. Bengio , and A. Courville , Deep learning. Genetic Programming and Evolvable Machines 19. (MIT Press, 2016).

[3] S. Sengupta , S. Basak , P. Saikia , et al., “A Review of Deep Learning With Special Emphasis on Architectures, Applications and Recent Trends,” Knowledge‐Based Systems 194 (2020): 105596, 10.1016/j.knosys.2020.105596.

[4] Council of Europe . Artificial Intelligence: Glossary (Council of Europe). Accessed, April 3rd, 2025, https://www.coe.int/en/web/artificial‐intelligence/glossary.

[5] N. Köbis , C. Starke , and I. Rahwan , “The Promise and Perils of Using Artificial Intelligence to Fight Corruption,” Nature Machine Intelligence 4, no. 5 (2022): 418–424, 10.1038/s42256-022-00489-1.

[6] F. Odilla , “Bots Against Corruption: Exploring the Benefits and Limitations of AI‐Based Anti‐Corruption Technology,” Crime, Law and Social Change 80, no. 4 (March 2023): 1–44, 10.1007/s10611-023-10091-0. 37359505 PMC10039446

[7] G. De Blasio , A. D'Ignazio , and M. Letta , “Predicting Corruption Crimes With Machine Learning,” in A Study for the Italian Municipalities (Sapienza University of Rome, DISS). Accessed, April 4th, 2025, https://ideas.repec.org/p/saq/wpaper/16‐20.html.

[8] F. J. López‐Iturriaga and I. P. Sanz , “Predicting Public Corruption With Neural Networks: An Analysis of Spanish Provinces,” Social Indicators Research 140, no. 3 (2018): 975–998, 10.1007/s11205-017-1802-2.

[9] J. Li , W.‐H. Chen , Q. Xu , N. Shah , J. C. Kohler , and T. K. Mackey , “Detection of Self‐Reported Experiences With Corruption on Twitter Using Unsupervised Machine Learning,” Social Sciences & Humanities Open 2, no. 1 (2020/01/01/2020): 100060, 10.1016/j.ssaho.2020.100060.

[10] G. M. Van De Ven , T. Tuytelaars , and A. S. Tolias , “Three Types of Incremental Learning,” Nature Machine Intelligence 4, no. 12 (2022): 1185–1197, 10.1038/s42256-022-00568-3. PMC9771807 36567959

[11] O. Onwujekwe , P. Agwu , C. Orjiakor , et al., “Corruption in Anglophone West Africa Health Systems: A Systematic Review of its Different Variants and the Factors That Sustain Them,” Health Policy and Planning 34, no. 7 (September 2019): 529–543, 10.1093/heapol/czz070. 31377775 PMC6788210

[12] H. Adobor and R. Yawson , “The Promise of Artificial Intelligence in Combating Public Corruption in the Emerging Economies: A Conceptual Framework,” Science and Public Policy 50, no. 3 (2023): 355–370, 10.1093/scipol/scac068.

[13] Open Government Partnership . Empowering Citizens as Watchdogs in Ukraine (Open Government Partnership), https://www.opengovpartnership.org/stories/lessons‐from‐reformers‐empowering‐citizens‐as‐watchdogs‐in‐ukraine/.

[14] S. S. Del , M. Gnaldi , and N. Salvini , “Sustainability and High‐Level Corruption in Healthcare Procurement: Profiles of Italian Contracting Authorities,” Socio‐Economic Planning Sciences 95 (2024): 101988, 10.1016/j.seps.2024.101988.

[15] M. J. García Rodríguez , V. Rodríguez‐Montequín , P. Ballesteros‐Pérez , P. E. D. Love , and R. Signor , “Collusion Detection in Public Procurement Auctions With Machine Learning Algorithms,” Automation in Construction 133 (2022): 104047, 10.1016/j.autcon.2021.104047.

[16] J. Wachs and J. Kertész , “A Network Approach to Cartel Detection in Public Auction Markets,” Scientific Reports 9, no. 1 (July 2019): 10818, 10.1038/s41598-019-47198-1. 31346221 PMC6658564

[17] K. Govindan , P. Jain , R. Kr. Singh , and R. Mishra , “Blockchain Technology as a Strategic Weapon to Bring Procurement 4.0 Truly Alive: Literature Review and Future Research Agenda,” Transportation Research Part E: Logistics and Transportation Review 181 (2024): 103352, 10.1016/j.tre.2023.103352.

[18] J. Gallego , G. Rivero , and J. Martínez , “Preventing Rather Than Punishing: An Early Warning Model of Malfeasance in Public Procurement,” International Journal of Forecasting 37, no. 1 (2021): 360–377, 10.1016/j.ijforecast.2020.06.006. 32836592 PMC7368425

[19] S. G. Graham , H. Soltani , and O. Isiaq , “Natural Language Processing for Legal Document Review: Categorising Deontic Modalities in Contracts,” Artificial Intelligence and Law 33, no. 1 (2025): 79–100, 10.1007/s10506-023-09379-2.

[20] P. A. Du , S. Bhattacharya , P. Beling , and E. Bowen , “Fraud Detection in Healthcare Claims Using Machine Learning: A Systematic Review,” Artificial Intelligence in Medicine 160 (2025): 103061, 10.1016/j.artmed.2024.103061. 39756221

[21] J. T. Hancock and T. M. Khoshgoftaar , “Gradient Boosted Decision Tree Algorithms for Medicare Fraud Detection,” SN Computer Science. 2, no. 4 (2021): 268, 10.1007/s42979-021-00655-z.

[22] H. De Meulemeester , F. De Smet , J. Van Dorst , E. Derroitte , and B. De Moor , “Explainable Unsupervised Anomaly Detection for Healthcare Insurance Data,” BMC Medical Informatics and Decision Making 25, no. 1 (2025): 14, 10.1186/s12911-024-02823-6. 39789541 PMC11720628

[23] J. S. Soo , “Artificial Intelligence in Anticorruption: Opportunities and Challenges,” GAB: The Global Anticorruption Blog. Accessed, April 4th, 2025, https://globalanticorruptionblog.com/2025/01/09/artificial‐intelligence‐in‐anticorruption‐opportunities‐and‐challenges/.

[24] A. Attaran , D. Barry , S. Basheer , et al., “How to Achieve International Action on Falsified and Substandard Medicines,” Bmj 345 (November 2012): e7381, 10.1136/bmj.e7381. 23149211

[25] T. K. Mackey and R. E. Cuomo , “An Interdisciplinary Review of Digital Technologies to Facilitate Anti‐Corruption, Transparency and Accountability in Medicines Procurement,” Global Health Action 13, no. sup1 (2020): 1695241, 10.1080/16549716.2019.1695241. 32194014 PMC7170358

[26] B. J. Ferdosi , M. A. Sakib , M. S. Islam , and J. Dhar , Identifying Counterfeit Medicine in Bangladesh Using Deep Learning (Springer, 2021), 46–55.

[27] M. Alsallal , M. S. Sharif , B. Al‐Ghzawi , and S. M. Mlkat al Mutoki , “A Machine Learning Technique to Detect Counterfeit Medicine Based on X‐Ray Fluorescence Analyser,” 2018 International Conference on Computing, Electronics & Communications Engineering (iCCECE) (2018): 118–122, 10.1109/iccecome.2018.8659110.

[28] T. A. Burt , N. Passas , and I. A. Kakadiaris , “AI‐SNIPS: A Platform for Network Intelligence‐Based Pharmaceutical Security,” Proceedings of the AAAI Conference on Artificial Intelligence 37, no. 13 (2023): 16407–16409, 10.1609/aaai.v37i13.27061.

[29] S. G. Finlayson , J. D. Bowers , J. Ito , J. L. Zittrain , A. L. Beam , and I. S. Kohane , “Adversarial Attacks on Medical Machine Learning,” Science 363, no. 6433 (March 2019): 1287–1289, 10.1126/science.aaw4399. 30898923 PMC7657648

[30] J. Zeng , “Artificial Intelligence and China's Authoritarian Governance,” International Affairs 96, no. 6 (2020): 1441–1459, 10.1093/ia/iiaa172.

[31] J. O'Donnell , How a New Type of AI Is Helping Police Skirt Facial Recognition Bans. Accessed, May 22nd, 2025, https://www.technologyreview.com/2025/05/12/1116295/how‐a‐new‐type‐of‐ai‐is‐helping‐police‐skirt‐facial‐recognition‐bans/.

[32] K. Farooq and B. J. Solowiej , “Artificial Intelligence in the Public Sector: Maximizing Opportunities, Managing Risks,” Equitable Growth, Finance and Institutions Insight (2021), http://documents.worldbank.org/curated/en/809611616042736565.

[33] F. Sbicca , Artificial Intelligence and Anti‐Corruption. Accessed, February 11th, 2025, https://ceur‐ws.org/Vol‐3762/481.pdf.

[34] T. Vian , B. Agnew , and K. McInnes , “Whistleblowing as an Anti‐corruption Strategy in Health and Pharmaceutical Organizations in Low‐ and Middle‐Income Countries: A Scoping Review,” Global Health Action 15, no. 1 (2022), 10.1080/16549716.2022.2140494. PMC9661981 36356311

[35] N. C. Köbis , C. Starke , and J. Edward‐Gill , The Corruption Risks of Artificial Intelligence (Transparency International). Accessed, March 20th, 2025, https://knowledgehub.transparency.org/assets/uploads/kproducts/The‐Corruption‐Risks‐of‐Artificial‐Intelligence.pdf.

[36] A. Amiruddin , S. Syamsuddin , S. J. Muntu , W. O. Helda , and G. M. D. P. Rahadi , “The Use of Artificial Intelligence (AI) in Internal Supervision to Reduce Fraud in the Public Procurement System in Indonesia,” ProBisnis: Jurnal Manajemen 15, no. 5 (2024): 769–780.

[37] D. Zinnbauer , Artificial Intelligence in Anti‐Corruption–A Timely Update on AI Technology (U4). Accessed, February 11th, 2025, www.u4.no/r/BR2501.

[38] Council of Europe . “Council of Europe Framework Convention on Artificial Intelligence and Human Rights, Democracy and the Rule of Law,” in Europe Co, Editor. Council of Europe Treaty Series (Vilnius2024).

[39] M. M. Ibrahimy , A. Norta , and P. Normak , “Blockchain‐Based Governance Models Supporting Corruption‐Transparency: A Systematic Literature Review,” Blockchain: Research and Applications 5, no. 2 (2024): 100186, 10.1016/j.bcra.2023.100186.

[40] K. Abbas , M. Afaq , T. Ahmed Khan , and W.‐C. Song , “A Blockchain and Machine Learning‐Based Drug Supply Chain Management and Recommendation System for Smart Pharmaceutical Industry,” Electronics 9, no. 5 (2020): 852, 10.3390/electronics9050852.

[41] D. Ressi , R. Romanello , C. Piazza , and S. Rossi , “AI‐Enhanced Blockchain Technology: A Review of Advancements and Opportunities,” Journal of Network and Computer Applications 225 (2024): 103858, 10.1016/j.jnca.2024.103858.

[42] G. Nicaise and D. Sejeroe Hausenkamph , Unlocking AI’s Potential in Anti‐corruption: Hype vs. Reality. (U4). Accessed, May 21st, 2025, https://www.u4.no/blog/unlocking‐ai‐s‐potential‐in‐anti‐corruption‐hype‐vs‐reality.

[43] IBM . What Are AI Agents? IBM. Accessed, May 25th, 2025, https://www.ibm.com/think/topics/ai‐agents.

[44] Operação Serenata de Amor . Por onde anda a Operação Serenata de Amor? Accessed, May 20th, 2025, https://apoia.se/serenata/contents/view/Por‐onde‐anda‐a‐Operacao‐Serenata‐de‐Amor‐w9FjFX‐az?page=1#0.

[45] Observatory of Public Sector Innovation . Robot Alice – Bid, Contract and Notice Analyser (OECD). Accessed, May 21st, 2025, https://oecd‐opsi.org/innovations/robot‐alice‐bid‐contract‐and‐notice‐analyser/.

[46] Pol.is . “Input Crowd, Output Meaning” Polis. Accessed, May 21st, 2025.

[47] vTaiwan . vTaiwan: Rethinking Democracy. Accessed, May 21st, 2025, https://info.vtaiwan.tw/.

[48] C. Starke , K. Kimon , R. Max , and N. Köbis , “Designing Algorithms Against Corruption: A Conjoint Study on Communicative Features to Encourage Intentions for Collective Action,” Journal of Information Technology & Politics (2025): 1–17, 10.1080/19331681.2025.2465326.

